# Nurturing Choices: How Sociodemographic Factors Affect Attitudes toward Breastfeeding

**DOI:** 10.1192/j.eurpsy.2025.2023

**Published:** 2025-08-26

**Authors:** A. Kondyli, I. Koutelekos, D. Briana, A. Zartaloudi

**Affiliations:** 1 MSc in General Pediatrics and Pediatric Subspecialties - Clinical Practice and Research, University of Athens; 2 University of West Attica; 3 University of Athens, Athens, Greece

## Abstract

**Introduction:**

The benefits of breastfeeding for mothers, infants, and society as a whole are well documented. These benefits appear to be linked to the duration of breastfeeding for both mother and child. Despite this knowledge, the rates of exclusive breastfeeding and continued breastfeeding at 6 and 12 months in Greece are exceptionally low.

**Objectives:**

To explore Greek parents’ attitudes towards breastfeeding according to their sociodemographic characteristics.

**Methods:**

A cross-sectional study was conducted using self-administered questionnaires completed by 862 parents—both mothers and fathers—who had received support from a private maternity and breastfeeding support center in Athens.

**Results:**

The choice of breastfeeding was positively correlated with parents’ higher educational level (p<0.001), normal delivery (p<0.001), residence in Athens or another urban area ((p=0.017), positive attitude towards breastfeeding during pregnancy (p<0.001) and for breastfeeding after 12 months (p<0.001), previous breastfeeding experience (p<0.001), not using a pacifier (p<0.001), introducing whole foods at 6 months ( p<0.001), co-sleeping with their baby (p<0.001) and not implementing sleep training (p<0.001). Accordingly, the duration of breastfeeding was positively associated with living in another urban area (p<0.001), and unemployment (p=0.009). Longer duration of breastfeeding showed children who were exclusively breastfed (p<0.001), who weaned naturally (p<0.001), who were born naturally (p<0.001), who did not take a pacifier (p<0.001), who started with whole foods ( p<0.001), who stayed longer in their parents’ room (p<0.001), who did not receive sleep training (p<0.001) and those whose parents were informed about breastfeeding (p<0.001).

**Conclusions:**

This study could serve as a foundation for more extensive research on breastfeeding. Findings can be utilized by health professionals to enhance their awareness, empathy, and effectiveness regarding issues related to breastfeeding.

**Disclosure of Interest:**

None Declared

